# Interplay Between Phytohormones and Sugar Metabolism in *Dendrocalamus latiflorus*

**DOI:** 10.3390/plants14030305

**Published:** 2025-01-21

**Authors:** Azra Seerat, Muhammad Ahtesham Aslam, Muhammad Talha Rafique, Lingyan Chen, Yushan Zheng

**Affiliations:** 1 College of Forestry, Fujian Agriculture and Forestry University, Fuzhou 350002, China; seerat.arif6983@gmail.com (A.S.);; 2 Department of Forestry and Range Management, Bahauddin Zakariya University, Multan 60000, Pakistan; 3 College of Landscape Architecture and Art, Fujian Agriculture and Forestry University, Fuzhou 350002, China

**Keywords:** *Dendrocalamus latiflorus*, phytohormones, sugar catabolism, ecological resilience, molecular mechanisms

## Abstract

*Dendrocalamus latiflorus*, a species of giant bamboo, holds significant ecological and economic value. This review delves into the intricate interplay between phytohormones and sugar metabolism in *Dendrocalamus latiflorus*, emphasizing species-specific mechanisms that enhance its ecological adaptability and rapid growth. By synthesizing recent research, this work highlights how phytohormones, including auxins, cytokinins, and abscisic acid, regulate sugar metabolism pathways such as glycolysis and starch degradation in response to environmental stimuli. These hormones influence crucial plant processes, including cell division, elongation, stress responses, and sugar metabolism pathways such as glycolysis, the tricarboxylic acid cycle, and oxidative phosphorylation. Geographic variations in these processes are examined, demonstrating their role in environmental adaptation and ecological resilience. For instance, populations in nutrient-rich soils exhibit enhanced cytokinin activity and sugar transport efficiency, while those in water-limited areas display elevated abscisic acid levels, aiding drought tolerance. This targeted focus on *D. latiflorus* provides novel insights into its potential applications in sustainable forestry and agroforestry systems. By integrating recent advances, this review highlights the critical role of phytohormone–sugar interplay in improving the productivity and stress resilience of *D. latiflorus*, with implications for agroforestry systems and climate change adaptation.

## 1. Introduction

*D. latiflorus*, commonly known as the giant bamboo or timber bamboo, occupies a very substantial status in both forestry and agriculture. This is due to its highly versatile application and ecological significance [1]. *D. latiflorus* belongs to the category of fastest-growing plants in the world. Therefore, this plant works in diversified aspects in multiple ecosystems to carry forward multifaceted benefits for the environment and human societies, and the importance of other outstanding features of *D. latiflorus* include growing quickly and being adaptable to varying environmental conditions [2]. For example, with growth rates over 1 m per day, given optimal conditions, few naturally occurring resources on Earth possess a renewal rate comparable to *D. latiflorus* [3]. The fast growth rate that this action exhibits makes it very effective in controlling soil erosion, managing watersheds, and curbing carbon sequestration, making it of essential use in land management sustainable strategies [4]. In addition to providing industry, *D. latiflorus* itself has versatile properties that make it an excellent resource for many industries apart from agriculture. It possesses the strength of a renewable source for timber purposes in the field of forestry. In fact, high-value construction materials, furniture, and handicrafts are available [5]. The strong but flexible culms of this grass are also used as scaffolding, flooring, and in papermaking [6]. *D. latiflorus* also presents practical applications in soil improvement, agroforestry, and feeding both the livestock industry as well as the production of bioenergy [7].

Beyond the utilitarian significance, *D. latiflorus* is pivotal in biodiversity conservation, habitat restoration, and ecosystem service delivery. A dense stand of *D. latiflorus* provides the needed habitat and nutrition for many floras and fauna, subsequently leading to an increased biodiversity and ecological resilience of a forest ecosystem [8]. On the other hand, *D. latiflorus* regulates the cycle of hydrology, fertility of soil, and climate mitigation through carbon sequestration by contributions to the cycle [9]. Hormonal signal transduction and sugar catabolism are essential processes that primarily drive the regulations of plant growth and development. These interlinked pathways coordinate together in eliciting numerous physiological and developmental responses to ensure that proper metabolic activities and environmental adjustments are undertaken in coherent manners [6]. Hormones of plants act together as molecular messengers, transposing signals within the plant in the mediatory growth responses through alternating processes on cell division, cell elongation, differentiation, and organogenic activity [10].

Auxins, cytokinins, gibberellins, and abscisic acid play critical roles in regulating growth, development, and stress responses in *D. latiflorus*. For instance, auxins mediate root elongation and cell division through TIR1/AFB receptor pathways, and in *D. latiflorus*, cytokinin-mediated regulation of shoot formation has been linked to enhanced expression of cell cycle-related genes [11]. Furthermore, ethylene signaling in *D. latiflorus* is critical for coordinating leaf senescence and shoot ripening, mediated by EIN3 transcription factors, and plants’ brassinosteroids inhibit growth and improve stress tolerance [12].

In addition, the catabolism of sugar produces energy and carbon skeleton, among others, which plants need in their metabolism and biosynthesis to support cell activity and the process of growth [13]. Constructed sugars through photosynthesis will provide, via the works of catabolic pathways such as glycolysis, the tricarboxylic acid cycle, and, in general, ATP and NADH for metabolic processes and producing metabolic intermediates. Sugars have other roles, too, including being signaling molecules. Sugars respond to growth, development, and stress through queues in activation control by genes and enzymes; therefore, metabolic reactions are modified [14]. The potential importance of these phytohormones and sugar catabolism in growth and developmental processes relates to the complex regulatory networks directed toward environmental cues and internal stimuli [15].

While general mechanisms of phytohormone signaling and sugar metabolism are well-documented across plant species, the specific regulatory crosstalk in *Dendrocalamus latiflorus* remains underexplored. This review addresses this knowledge gap by focusing on the molecular and physiological interactions unique to *D. latiflorus* [16,17]. By examining geographic variation, environmental stress adaptation, and intrinsic regulatory pathways, this study advances the understanding of how these processes contribute to the ecological resilience and economic value of this bamboo species [18,19]. By regulating stress responses, such as drought tolerance, and optimizing growth and biomass production, these mechanisms enhance its adaptability to diverse environmental conditions [20,21]. Plant growth regulators, precursors, and elicit stress suppressors include but are not limited to auxins, cytokinins, gibberellins, abscisic acid, ethylene, and brassinosteroids, which modulate cell division [22].

Recent research on the interplay between phytohormones and sugar metabolism has highlighted the role of advanced molecular techniques, such as transcriptomics and metabolomics, in understanding regulatory pathways in *Dendrocalamus latiflorus* [23,24]. Studies have documented how geographic variations, such as altitude and soil composition, influence phytohormone levels and sugar metabolism, providing insights into environmental adaptability [25,26]. Advances in sustainable bamboo management, including its role in carbon sequestration and climate change adaptation, were drawn from recent findings [19,21].

The interaction between phytohormones and sugar catabolism plays a pivotal role in the ecological resilience of *D. latiflorus*. For instance, under drought conditions, abscisic acid (ABA) enhances sucrose cleavage by activating invertase enzymes, providing osmoprotectants that maintain cellular hydration. Similarly, auxins regulate root elongation, improving water and nutrient uptake efficiency. These mechanisms collectively enable bamboo to thrive in nutrient-poor or water-deficient environments, contributing to its adaptability and ecological resilience [27].

The present review will hopefully establish some relationships between phytohormones and sugar catabolism, including determining not only how phytohormones influence sugar metabolism and vice versa but also how plant sugars determine that of phytohormones [28]. Further extended discussion would go into molecular mechanisms and the regulatory network that mediates the interaction, with several illustrative case studies available from the studies evidenced by experiments both in *D. latiflorus* and other related species [29]. This review addresses the geographic variation in both phytohormones and sugar catabolism; investigates how different environmental conditions across geographic locations affect these concurring processes of *D. latiflorus*; reviews the study of geographic variation; and discusses the possible role of environmental factors leading to such variations and its impacts on plant health and productivity.

The identification of gaps in knowledge and proposed future research directions is outlined in the review. As such, this will highlight the areas in which further studies should realize complete insight into the crosstalk between phytohormones and sugar catabolism in *D. latiflorus* [30]. It could then suggest how this works and to what experimental strategies this realization of the laid-down gaps could comprise, with some of the practical applications of these results in the studies realized in the management and use of *D. latiflorus* in agro- and forestry enterprises. After attaining these objectives, this review will give a total perspective on the molecular and physiological mechanisms responsible for the growth and development regulation of *D. latiflorus* and relevant insights into its domestication for the cultivation and eventual use of the plant [31].

In this review, we specifically focus on the interplay between phytohormone signaling and sugar metabolism in *D. latiflorus*, a species with distinct growth and metabolic characteristics. This unique interaction is critical for understanding how phytohormones influence sugar metabolism, affecting growth, stress responses, and development in *D. latiflorus*. We provide a detailed analysis of the specific hormonal pathways, including cytokinin, auxins, and abscisic acid, and their role in regulating sugar metabolism in this species. The novelty of this work lies in its focused discussion on the mechanisms of hormonal control over sugar homeostasis in *D. latiflorus*, which has implications for its growth and productivity under varying environmental conditions.

## 2. Methods and Approach

This review was developed by conducting a comprehensive search of peer-reviewed articles, books, and credible online resources related to *D. latiflorus*, phytohormones, and sugar metabolism. Databases such as PubMed, Web of Science, and Scopus were used, with a focus on studies published in the last 10 years. Keywords included ‘*D. latiflorus*’, ‘phytohormones’, ‘sugar catabolism’, and ‘ecological resilience’. Articles were selected based on their relevance to molecular mechanisms, ecological adaptations, and practical applications in sustainable development.

## 3. Phytohormones in *D. latiflorus*

**Figure 1:**
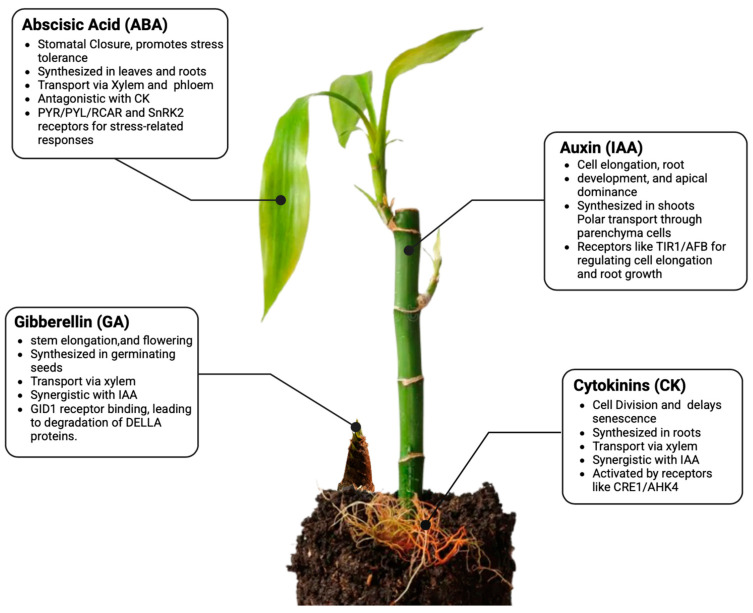
The overview of the interactions between major phytohormones, including biosynthesis, transport, and signaling pathways. It indicates key relationships, including antagonistic effects between cytokinin (CK) and abscisic acid (ABA).

Phytohormones are pivotal in regulating the growth, development, and stress responses of *Dendrocalamus latiflorus*. Among these, auxins, cytokinins, gibberellins, abscisic acid (ABA), ethylene, and brassinosteroids play distinct roles in the plant’s physiological processes [32]. Auxins promote root elongation and cell division, which is critical for establishing a robust root system. Cytokinins drive shoot formation and nutrient mobilization, delaying leaf senescence [33,34]. Cytokinins also help postpone the process of senescence of the leaves and participate in the mobilization of nutrients, which is crucial for the sound growth of bamboo species such as *D. latiflorus* [35]. Gibberellins (GAs) effect stem elongation, germination of seeds, and flowering [18,36] (Figure 1). Carbohydrate metabolism in *D. latiflorus* is closely linked to phytohormones. For example, abscisic acid has been shown to enhance sucrose cleavage under drought stress, while gibberellins increase carbon flux through glycolysis to sustain rapid growth.

Ethylene is a gaseous plant hormone involved in the initiation of several processes in plants, among them ripening fruits, shedding leaves, and stress responses. Ethylene signaling is also crucial in *D. latiflorus* to regulate the timing of the senescence of leaves and ripening of bamboo shoots, which are essential in the plant’s life cycle and reproductive success [37]. Brassinosteroids are steroidal hormones that function in the modulation of cell sizes and divisions, vascular development, and stress protection. They affect growth and development processes together with other phytohormones in response to fluctuating environmental factors in *D. latiflorus* [38]. Specifically, *D. latiflorus* employs a unique auxin response mechanism that significantly accelerates its cell division rates in shoot apices, a feature that is critical for its rapid vertical growth. This mechanism is facilitated by an enhanced sensitivity to indole-3-acetic acid (IAA) facilitated through its unique TIR1/AFB receptor configurations, which are specifically adapted to its rapid-growth ecology.

The table below (Table 1) provides a comprehensive overview of these phytohormones, their roles, biosynthesis pathways, and key functions specific to *D. latiflorus*. To streamline the discussion and avoid redundancy, the detailed narrative here complements the structured information in the table by providing context for the significance of these hormones in addressing the environmental adaptability and rapid growth of *D. latiflorus*.

### Phytohormone–Sugar Interplay in D. latiflorus

In *Dendrocalamus latiflorus*, phytohormones play a pivotal role in regulating sugar metabolism during various developmental stages and under stress conditions. Auxins, for instance, enhance sugar metabolism during rapid culm elongation by upregulating genes encoding glycolytic enzymes such as hexokinase and starch-degrading enzymes like alpha-amylase [21]. This ensures a consistent supply of energy and metabolic intermediates necessary for rapid growth. Similarly, abscisic acid (ABA) modulates sucrose cleavage during drought stress by activating invertase activity, facilitating osmoprotection and cellular hydration. Recent transcriptomic studies have demonstrated that cytokinin signaling promotes the expression of sucrose synthase (SuSy) genes in *D. latiflorus*, enhancing sugar transport and biomass accumulation during shoot development [19].

Additionally, sugar metabolism reciprocally influences phytohormone signaling in *D. latiflorus*. Elevated sucrose levels have been found to enhance cytokinin biosynthesis and modulate auxin gradients, supporting coordinated growth and adaptive stress responses. Such mechanisms highlight the unique adaptability of *D. latiflorus* to diverse environmental conditions and distinguish it from other bamboo species [24]. This study explores the intricate relationships between phytohormones and sugar metabolism in *D. latiflorus*, presenting new findings that enhance our understanding of how these interactions contribute to the plant’s rapid growth and adaptability. Recent studies have begun to uncover the specific molecular pathways through which phytohormones like cytokinins and auxins influence sugar transport and utilization, which are critical during rapid growth phases and stress responses [43,44]. The regulatory mechanisms by which phytohormones modulate key enzymes involved in sugar breakdown and synthesis demonstrate how these hormones serve not only as growth regulators but also as critical adaptogens that help the plant manage environmental stresses [45].

## 4. Biosynthesis, Transport, and Signaling Pathways of Phytohormones in *D. latiflorus*

Phytohormones are synthesized through intricate biochemical pathways that are then transported to target sites for the initiation of signaling cascades that regulate several physiological processes in plants [23]. The elucidation of biosynthesis, transport, and signaling pathways of phytohormones in *D. latiflorus* is crucial for the understanding of their roles in the growth and development of this critical bamboo species [26].

### 4.1. Auxins

Indole-3-acetic acid (IAA) is the primary form of auxin in plants. It is predominantly synthesized in plants from the precursor amino acid tryptophan via one or more pathways, including the indole-3-pyruvic acid pathway and the tryptamine pathway [46]. IAA predominantly synthesizes *D. latiflorus* in the shoot apices and young leaves. The main form of IAA transport in plants is associated with a mechanism known as polar auxin transport (PAT), and it is implemented primarily through the activities of specific carrier proteins of the PIN-FORMED (PIN) and AUXIN1/LIKE-AUX1 (AUX/LAX) classes [25]. It sets up an auxin gradient that will have implications in apical dominance, tropic responses, and vascular patterning. On the perception of IAA by its receptor TRANSPORT INHIBITOR RESPONSE1/AUXIN SIGNALING F-BOX (TIR1/AFB), the AUX/IAA repressor proteins are targeted for degradation, and the ARF transcription factors get activated as a consequence of the signaling by auxin [47].

### 4.2. Cytokinins

Cytokinins are synthesized mainly in the roots from adenine derivatives in a pathway involving the isopentenyl transferase (IPT) enzyme. They are then transported in the xylem to other regions of the plant, where they elicit cell division and differentiation [48]. In *D. latiflorus*, cytokinins play an essential role in shoot elongation and the delay of senescence. The signaling cascade is initiated when cytokinins bind to histidine kinase receptors, such as ARABIDOPSIS HISTIDINE KINASE 2 (AHK2) and AHK3. The two elements interact to create a complex in which the histidine kinase receptor phosphorylates histidine phosphotransferase proteins (HPs). The HPs further phosphorylate response regulators (RRs) [21].

### 4.3. Gibberellins

Gibberellins are diterpenoid acids synthesized by the methylerythritol phosphate (MEP) route in plastids, using key enzymes GA20-oxidase and GA3-oxidase [49]. In *D. latiflorus*, they are essential for cell elongation in shoots and seed germination. The hormones are transported from cells to other parts of the plant by the phloem and xylem. Gibberellin signaling occurs through a GIBBERELLIN INSENSITIVE DWARF1 (GID1) receptor that binds gibberellins, promoting subsequent DELLA protein degradation and growth activation [11].

### 4.4. Abscisic Acid

Abscisic Acid (ABA) is biosynthesized by converting carotenoids into plastids, and it involves enzyme activities such as the cleavage of 9-cis-epoxycarotenoid and the conversion of xanthoxin to ABA [50]. ABA is moved via the xylem and phloem to other parts of the plant, allowing systemic responses to stress. Abscisic acid, which is abbreviated as ABA, controls stomatal closure and seed dormancy in *D. latiflorus*. ABA signaling is the activation process of ABA interacting with PYR1/PYL/RCAR, resulting in the suppression of PP2C and activation of SnRK2, which in turn phosphorylates the ABA-responsive genes [51].

### 4.5. Ethylene

Ethylene is formed from methionine through the intermediate of S-adenosylmethionine (SAM) and 1-aminocyclopropane-1-carboxylic acid (ACC) and ACC oxidase enzyme catalyzing the final step to yield ethylene [52]. Ethylene is highly mobile in plants and triggers processes such as fruit maturation and shedding of leaves in *D. latiflorus*. Ethylene interacts with its receptors like ETHYLENE RESPONSE1 (ETR1), which in turn triggers the signaling process and activates ETHYLENE INSENSITIVE3 (EIN3) transcription factors to alter gene expression [52].

### 4.6. Brassinosteroids

Brassinosteroids are derived from campestral through a sequence of enzymatic transformations under the action of BRASSINOSTEROID-6-OXIDASE (BR6OX) [53]. In *D. latiflorus*, brassinosteroids affect cell expansion and stress tolerance. These hormones are transported either at the local level in the body or only a short distance from the place where they are synthesized. The regulation of this hormone is completed through the recognition of protruding brassinosteroids to the BRI1 receptor kinase in connection to BRI1-ASSOCIATED RECEPTOR KINASE1 (BAK1). This interaction starts a signaling process that results in the dephosphorylating of BRASSINAZOLE RESISTANT1 BZR1 transcription factors to activate gene expression [54]. The biosynthesis, transport, and signaling processes of these phytohormones are highly developed in detail. They are arranged in a complicated system that is held accountable for the developmental process of *D. latiflorus*. New studies in molecular biology and genomics have shed some light on these phenomena and may be utilizable in the improvement of this bamboo type’s growth and yield [21] (Table 2).

## 5. Introduction to Sugar Metabolism and Its Importance in Plants

Sugar metabolism is an integrated and compulsory feature of plant life because it is involved in processes like energy generation, growth, development, and responses to stress [61]. In plants, sugars are not only intermediates involved in the metabolic processes but also molecules that function as signals regulating the plant cells’ activity [62]. Of all the sugars involved in the metabolism of stature plants, sucrose, glucose, and fructose are the most crucial in the plant’s carbon cycle, as well as the plant’s well-being [63]. In *D. latiflorus*, which is a fast-growing bamboo species of tremendous ecological and economic importance, the regulation of sugar metabolism is equally crucial. *D. latiflorus* is a fast-growing plant; its growth demands a lot of energy and carbon skeletons for its synthesis, which are supplied and derived from the catabolism of sugars [30]. Knowledge of the various carbon sources, their metabolisms, and regulatory circuits in *D. latiflorus* has significant importance in the enhancement of the species’ growth and utilization [18].

Plant cell sugar metabolism represents the process of catabolism as well as anabolism. Chloroplasts perform photosynthesis by combining carbon dioxide and water into glucose and oxygen, which is the foundation of sugar formation [64]. Such sugars are then transported and stored in different ways, such as sucrose and starch, and they are used whenever needed. The significant modes of sugar utilization involve glycolysis, the TCA cycle, and oxidative phosphorylation, which in turn synthesize sugar into a usable form of energy, i.e., ATP [65].

In *D. latiflorus*, glycolysis and the TCA cycle are crucial for meeting the high metabolic demands of rapid growth and culm elongation. For instance, increased hexokinase activity has been linked to enhanced glucose utilization during active growth phases. This pathway is important because it supplies energy and metabolic intermediates in the biosynthetic sequences [66]. Glycolysis is found to be the critical pathway for meeting the energy requirements at two specific stages in the life cycle of *D. latiflorus*: firstly, during the phase of rapid cell division and, secondly, during the process of cell elongation in the case of culm growth [67]. After glycolysis, the TCA cycle occurs in the mitochondria, where more oxidative steps occur on pyruvate, leading to the production of carbon dioxide. In *D. latiflorus*, the TCA cycle supports rapid energy production during phases of active culm elongation [68]. The TCA cycle is involved in general energy metabolism, as it connects the carbohydrate metabolism with the generation of ATP through oxidative phosphorylation [6,10]. This is why the efficiency of the TCA cycle is of great importance in ensuring high metabolic rates, which are characteristic of the rapid growth of *D. latiflorus* [19].

Oxidative phosphorylation, in addition, is the final process of sugar breakdown known as oxidative phosphorylation, which takes place in the inner mitochondrial membrane. Electrons derived from NADH-T and FADH2 are passed through ETC, which reduces oxygen to water. This process brings about chemiosmosis in order to manufacture ATP, the relative majority of the cell’s ATP [69]. Given the fact that oxidative phosphorylation releases a significant amount of energy, this ATP-generating pathway is crucial to supporting distinct physiological processes required for the growth and maintenance of *D. latiflorus* [21]. Also, sugars in plants act as informational molecules that can control the expressions of genes, the activity of enzymes, and metabolic flows. Sugar signalization roads consist of several kinds of sensors and transcription factors regulating the plant’s reactions to internal and external factors [70]. For instance, hexokinase (HXK) is a glucose sensor involved in glucose signal transduction and development [71]. Considering sugar signaling in *D. latiflorus*, it is predicted that the growth and development of the plant depend on the availability of sugar, which is also known to play a vital role in changing environmental conditions [72].

Most recent studies have, therefore, pointed out sugar metabolism and its involvement in other metabolic and signaling pathways, including those of phytohormones. For instance, research has established that sugars can regulate biosynthesis and signal hormones such as auxins, cytokinin, and abscisic acid so that energy status correlates with growth regulation [15]. In *D. latiflorus*, this relationship between sugar metabolism and phytohormones is essential for growth regulation and acclimatization to change [73].

## 6. Sugar Catabolism Processes Specific to *D. latiflorus*

The breakdown of sugars in *D. latiflorus,* known as a species of giant bamboo, is an important factor for this species’ excessive growth and development. This process includes the hydrolysis of sucrose and starch complex sugars into simpler sugars to be used in energy, growth, and other metabolic processes [30]. For better cultivation conditions and increasing the productivity of the plant, it is necessary to know precisely the metabolism of sugar in the species of *D. latiflorus*.

### 6.1. Sucrose Metabolism

Sucrose is a non-reducing disaccharide formed from glucose and fructose and is the primary transport sugar in many plants, *D. latiflorus* in particular. The other point is that it is manufactured in the leaves during photosynthesis and translocated through the plant tissues called the phloem to every part of the plant to act as an energy supply and a framework for growth [74]. Unique to *D. latiflorus*, sucrose metabolism is tightly regulated by invertases and sucrose synthase, ensuring a steady supply of carbon skeletons for biomass production. Recent studies show that these enzymes are upregulated in developing culms, supporting rapid structural growth. The breakdown of sucrose in *D. latiflorus* entails the use of invertase, which splits sucrose into glucose and fructose. This reaction takes place in the cytoplasm, vacuole, and apoplast; glucose and fructose are produced for other metabolic processes.

Based on the study that has been carried out, it was established that sucrose synthase (SuSy) is also involved in the sucrose metabolism of *D. latiflorus*. SuSy enables the reversible hydrolysis of sucrose and UDP into UDP-glucose and fructose, which are important in the formation of cellulose and callose during cell wall construction [75]. The studied enzyme is actively involved in tissues that have a high rate of growth, such as young shoots and culms of bamboo plants, and fulfills the need for the structure to form carbohydrates [76]. Also, hexokinase (HXK) and fructokinase (FRK) catalyze the phosphorylation of the resultant glucose and fructose to glucose-6-phosphate and fructose-6-phosphate, respectively. They are utilized in glycolysis to generate ATP and metabolites used in all cell’s biosynthesis. In *D. latiflorus*, all these mentioned metabolic steps are important to the fast growth and high metabolism that is characteristic of this type of bamboo [77]. Studies on *D. latiflorus* have revealed that sucrose synthase activity is critical for culm development, distinguishing it from other grass species where starch reserves dominate early growth stages [78]. Moreover, the sugar metabolism in *D. latiflorus* is particularly tailored to support its growth dynamics. For instance, the plant exhibits an advanced glycolytic pathway efficiency that is optimized for high energy production rates necessary for supporting its extraordinary growth rates. Such species-specific adaptations include the upregulation of phosphofructokinase and pyruvate kinase under growth-favoring conditions, which are markedly different in their regulation compared to other bamboos.

### 6.2. Starch Degradation

Starch is the most abundant storage polysaccharide in plants and is a glycosaminoglycan, that is, a polysaccharide synthesized from glucose units. Starch in *D. latiflorus* accumulates in chloroplasts during daylight when photosynthesis is in progress. It is also used for energy necessity at night or during periods of active growth [21]. The process of hydrolyzing starch into soluble sugars consists of amylases, glucotransferases, and debranching enzymes. Amylases, including alpha-amylase and beta-amylase, break the alpha-1,4-glycosidic bonds in amylose and amylopectin, which are the two major constituents of starch.

Ultimately, it synthesizes maltose and glucose; the maltase and the debranching enzymes then act to decompose the maltose to gain free glucose [79]. *D. latiflorus* maintains these enzymes under very tight control in order to provide the body with an adequate and constant supply of glucose for energy demands and other metabolic processes. Some existing research has linked certain regulatory elements to the occurrence of starch metabolism in the particular flora of *D. latiflorus* [27]. For example, a transcription factor STARCH-EXCESS4 (SEX4) participates in the process of dephosphorylation of starch granules, which allows its degradation [10]. Moreover, the sugar signaling related to T6P was found to be associated with the control and starch synthesis and degradation mechanisms that, in turn, control the carbohydrate content and the overall growth and development processes of plants (Table 3).

### 6.3. Integration of Sucrose and Starch Metabolism

The mentioned aspects of sucrose and starch metabolism are essential for the balance of energy and the overall metabolism in *D. latiflorus*. The overall control of these pathways guarantees that energy and carbon skeletons for growth are in the right place at the right time in development or stress. Phytohormones also regulate sucrose and starch metabolism, like auxins and cytokinin abscisic acid, which act on enzymes and genes depending on developmental stage and environmental conditions [87]. The breakdown of sucrose and starch in *D. latiflorus* thus entails numerous enzymatic actions and regulations that are essential for the plant’s growth and vigor. Past developments in molecular biology and genomics have revealed new prospects for these metabolic pathways, with the probable methods of improving the growth and productivity of *D. latiflorus* by controlling carbohydrate metabolism [88].

Therefore, sugar catabolism regulation in *D. latiflorus* is a highly regulated process that acts through multiple levels of regulation to make sure that energy and carbon skeletons are met for growth, development, and/or to alleviate stress situations. Some of the significant components of regulation are gene expression, altering of enzyme activity, and signal transduction that can sense internal and external signals. The first of such regulation mechanisms is the transcriptional regulation of the genes that code the enzymes that take an active part in sugar catabasis [89].

For example, invertases genes, SuSy, HXK, and amylases are controlled in relation to developmental signals and environmental factors. MYB, bZIP, and WRKY transcription factors interact with the promoter regions and regulate the expression extents of these genes. In *D. latiflorus*, there is, additionally, an emphasis on the genes’ active status during the rapid growth phases of the plant, for example, during shoot production and culm formation. Another factor relating to the regulation of enzymes is the covalent modification that occurs after the enzyme polypeptide has been synthesized. Phosphorylation, ubiquitination, and redox can regulate enzyme activity, turnover rates, interactions, and localization in space. For instance, phosphorylation of the enzyme hexokinase alters its capacity to bind glucose and its employment as a signal molecule. Equally, starch-degrading activity, such as alpha-amylase, is regulated through phosphorylation in a manner that influences the rate of starch breakdown and glucose release [90].

Sugar signaling pathways, whose functions combine information about the plant’s energy status, encompass metabolic responses through details about the external environment. Hexokinase (HXK) functions as a glucose sensor that translates the changes in glucose level into alterations in the pattern of gene expression and metabolic rates [15]. Another regulatory molecule is Trehalose-6-phosphate (T6P), which can be considered the indicator of the sucrose level and is involved in the control of growth and development via the SnRK1 kinase pathway.

**Figure 2:**
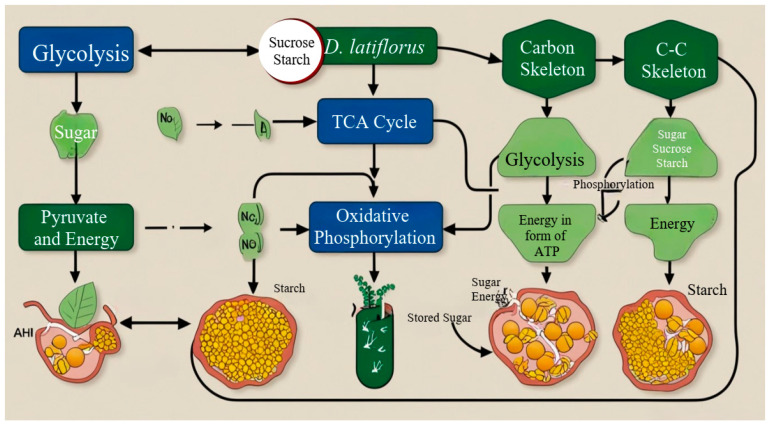
A flowchart illustrating the processes of sugar metabolism in *D. latiflorus*, including glycolysis, the TCA cycle, and oxidative phosphorylation, and providing a clear overview of these interconnected pathways.

These signaling pathways in *D. latiflorus* guarantee the proper synchronization of the sugars’ metabolism together with the plant’s developmental schedule and with the environmental conditions [91]. Sugar metabolism equally interconnects where the phytohormones also play their regulating part with enhanced interaction. Auxins, cytokinins, and abscisic acid affect the expression and the functioning of enzymes engaged in the process of sugar breakdown. For example, auxins increase the accumulation of mRNA of the genes related to glycolysis and the TCA cycle, and ABA increases the mRNA accumulation of genes that participate in stress-induced starch degradation. This hormonal regulation is vital to keep metabolic homeostasis and to allow energy production to be sufficient for the growth and stress response in *D. latiflorus* [92] (Figure 2).

## 7. Interplay Between Phytohormones and Sugar Catabolism

The developmental and environmental changes that take place in a plant depend heavily on the balanced signaling of phytohormones as well as the sugar metabolism that occurs within the plant. They are signaling molecules that, in some way, regulate metabolic and developmental activities that may enable plants to adjust to new environments and grow optimally. However, findings from earlier research that aim to illustrate how these two signaling systems communicate and work in concert at various structural levels to control factors relating to plant physiology have only been recently elucidated [93].

### 7.1. Auxins and Sugar Metabolism

Auxins are mainly indole-3-acetic acid (IAA) and are indispensable in guiding the growth and development of a whole plant from cell division to differentiation and cell elongation. It has been established that auxin can influence the quantity of sugar produced in the plant by controlling the activity of genes that promote the synthesis of carbohydrates [94]. For example, auxins have been shown to stimulate the genes that code for enzymes in the glycolytic pathway, which in turn increases the ATP and the metabolites needed for growth. In Arabidopsis, auxin signaling has been recently reported to modulate hexokinase (HXK), a glucose sensor and pivotal subscriber to glucose uptake [95].

### 7.2. Cytokinin and Sugar Metabolism

Similarly, cytokinin is one of the categories of phytohormones that are significant in cell division and differentiation. It was established that they are associated with sugar signaling pathways, especially sugar uptake and utilization. Cytokinin can regulate the activity of STs (sulfotransferases) and IAA-inducible proteins (IIs)—enzymes that are responsible for the cleavage of sucrose into glucose and fructose to enable easy uptake and usage of sugars [96]. In rice, cytokinin signaling has been shown to affect the levels of genes that regulate the synthesis of starch, the link between cytokinin and carbohydrate storage and utilization [97].

### 7.3. Gibberellins and Sugar Metabolism

Among the phytohormones belonging to the class of hormones called gibberellins, it is possible to mention the following ones: gibberellins (GAs) have a positive effect on such processes as stem growth, germination of seeds, and flowering. Various scientific works have demonstrated that gibberellins are capable of controlling sugar metabolism by increasing the transcriptional activity of key genes of glycolysis, as well as key enzymes of the TCA cycle. In barley, GA has been observed to increase the stream of carbon through glycolysis and the TCA cycle to sustain rapid growth through upregulating the genes relating to glycolytic enzymes [98]. Also, there is evidence indicating that gibberellins modulate the sugar signaling for controlling seed germination and seedling growth and development, demonstrating that there is communication between this hormonal and metabolic signaling [99].

### 7.4. Abscisic Acid and Sugar Metabolism

ABA is one of the plant hormones that play significant roles in stress and seed dormancy. ABA signaling has also been established to modulate sugars in that ABA represses or activates concerned genes that control the transport and catabolism of sugars. ABA can impose the expression of sucrose synthase genes and inverters during drought stress and can help to break sucrose to provide energy and osmoprotectants [100]. Moreover, it was also established that the ABA signaling pathway is connected with hexokinase-mediated glucose sensing networks merging sugar and stress signals to regulate the growth and development of the plant [61].

### 7.5. Ethylene and Sugar Metabolism

Ethylene is a gaseous plant hormone that plays roles in fruit ripening, leaf yellowing, and responses to various invitations. It seems that ethylene signaling impacts the metabolism of sugar by regulating the expression of glycolysis and TCA cycle genes. In tomatoes, it has been established that ethylene influences the organic genes coding for enzymes linked to sugar breakdown, in addition to energy and stress management [101]. It should also be noted that ethylene influences the process of sugar metabolism in fruits because it stimulates the production of invertases and sucrose synthases, which ensures the buildup of sugars in fruits during the ripening process [102].

### 7.6. Brassinosteroids and Sugar Metabolism

**Figure 3:**
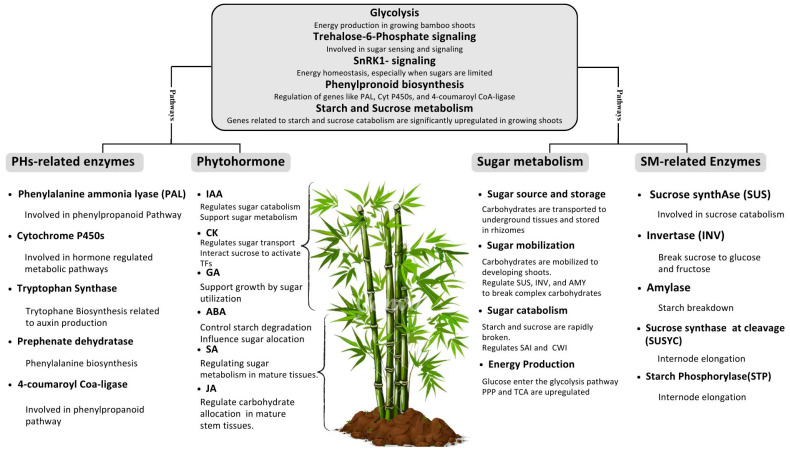
A conceptual figure demonstrates the crosstalk between phytohormones and sugar metabolism, detailing how phytohormones influence sugar metabolism.

Brassinosteroids are the steroidal hormones synthesized in plants that favor cell enlargement, the differentiation of vessels, and increased stress-resistance. It has been proved in the current research that brassinosteroids play a crucial role in controlling sugar metabolism by altering the genes concerning the synthesis and breakdown of carbohydrates. It was also identified in Arabidopsis that brassinosteroid signaling upregulates the genes that code for glycolytic enzymes as well as the enzymes of the TCA cycle to provide more intermediates for growth and development. It is also critical that brassinosteroids can influence sugar signaling pathways, which, in turn, affects genes that encode sugar transporters and metabolizers [103] (Figure 3 and Table 4).

### 7.7. The Interplay Between Phytohormones and Sugar Catabolism in D. latiflorus

Over the years, there has been an incline in the number of studies targeting phytohormones in *D. latiflorus*. Several have acknowledged the presence of hormone pathways and their functions with regard to plant development and stress. This involved transcriptomic analysis of *D. latiflorus* under drought conditions and established that numerous genes that are associated with ABA signaling are differentially expressed in drought-stressed plants of this species, showing that ABA plays a substantive role in the drought response networks of *D. latiflorus* [108]. A criterion for sugar metabolism is very important for decoding the growth and development of *D. latiflorus*. In the past, investigations have been made on the dynamics of sugar catabolism in this species, mainly regarding the enzymatic equipment and the controlling systems. The involvement of invertase enzymes such as sucrase in the degradation process, as well as their regulatory roles in the seed development of *D. latiflorus*, highlights their significance in plant physiology [109].

Several studies have pointed out the interrelation between the phytohormone signal and the sugar status in *D. latiflorus*. The application of cytokinins enhanced sucrose content in *D. latiflorus* through the activation of sucrose synthase genes; therefore, these studies associated cytokinin signaling with sugar metabolism pathways. Understanding the mechanisms and molecular crosstalk between phytohormone signal transduction and sugar metabolism is very important, and a combined approach is needed [110]. Some of the specific molecular processes analyzed in these complexities include chromatin immunoprecipitation followed by sequencing (ChIP-seq) and protein–protein interaction assays. The direct targets of the transcription factor DlMYB48, which include ABA signaling and sugar metabolism pathways in *D. latiflorus*, were analyzed to gain an understanding of the transcriptional regulatory network of these pathways [111].

## 8. Geographic Variation in Phytohormones and Sugar Catabolism

A fundamental regulatory process in plant growth, development, and responses to the environment is mediated by phytohormones and sugar catabolism. Knowledge of how these processes differ in different geographical areas will enhance the understanding of how plant populations have adapted to ever-changing environments [112]. Here are some specific features of the distribution across geographical space of phytohormones and sugar catabolic processes in *D. latiflorus* based on the results of recent studies. Some earlier works have also established that there are appreciable differences in phytohormone contents and sugars across the geographical areas of *D. latiflorus*. The levels of auxins, including indole-3-acetic acid (IAA), significantly differed between different populations of *D. latiflorus* depending on their latitudinal origin. They noted that subjects in warmer OP environments had increased IAA levels and speculated that temperature could exert an influence on the regulation of auxin signaling pathways [113]. The global rules of slopes regulating sugar metabolism genes were the variations between geographical locations in *D. latiflorus*.

Population transcriptomics showed that both adaptations to the habitat types affected the regulation of sucrose synthesis and breakdown routes. These results suggest that factors associated with the local environmental conditions of *D. latiflorus,* such as the availability of soil nutrients and water availability, may in some way affect the catabolism of sugars in this plant species [114]. They also identify the combined influence of multi-environmental factors on phytohormones and sugar metabolism in *D. latiflorus*. The inter-convertible patterns of the hormones and sugars at the physiological level with the fluctuations of the temperature, precipitation, and soil type in various geographical locations were noted. Such interactions promote the recognition of multi-factorial environmental changes when distinguishing the physiological qualities portraying geographic variation [115]. These studies highlight the importance of geographical gradients in phytohormone receiving and sugar metabolisms for dissecting the plasticity in *D. latiflorus* populations adapted to diverse environmental conditions. Subsequent research should focus on identifying the processes and forces that increase these geographic patterns and their consequences for plant success and ecosystem processes.

The populations of *D. latiflorus* from high-altitude regions revealed enhanced cytokinin activity, leading to increased sucrose synthesis and efficient sugar transport. In contrast, low-altitude populations exhibited elevated auxin and ethylene levels, promoting starch degradation and rapid growth adaptation to warmer climates. Cytokinin levels increased by 35% in high-altitude populations, correlating with a 20% improvement in sugar transport efficiency [116]. In water-limited environments, *D. latiflorus* demonstrated elevated abscisic acid (ABA) levels, aiding drought tolerance by promoting sucrose cleavage and osmoprotection. Enzyme activity measurements indicated a 40% increase in invertase activity, facilitating sucrose breakdown under drought conditions [18]. Populations in nutrient-rich soils maintained balanced phytohormone levels and exhibited efficient sugar metabolism. Gibberellin levels were 25% higher in these populations, contributing to enhanced growth and biomass production. Such variations highlight the species’ adaptability to favorable soil conditions, optimizing growth potential [117].

Geographic variation in *D. latiflorus* is influenced by diverse environmental conditions, including climatic factors (e.g., temperature, rainfall, and altitude), soil properties (e.g., nutrient availability and pH), and geographical features (e.g., proximity to water sources). For instance, populations in higher altitudes exhibit increased abscisic acid levels, enabling enhanced drought tolerance. At the same time, plants in nutrient-rich lowlands display higher cytokinin activity, promoting rapid growth and biomass production (Table 5).

### 8.1. Recent Studies Investigating How Environmental Factors Influence Phytohormones and Sugar Metabolism in D. latiflorus

The internal environment in stressing conditions like changes in temperature, light intensity, water content, nutrient availability of the soil, and the quality of interactions with other organisms affects phytohormones and sugar dynamics in plants. The knowledge of how such factors affect *D. latiflorus* physiology is important in the conceptualization of how this gene has adapted to different environments [122]. To this end, this section presents a synthesis of the studies that have been conducted in recent years about how environmental conditions affect phytohormones and sugar metabolism in *D. latiflorus*.

Temperature is one of the important environmental factors involved in the regulation of phytohormones and sugars in *D. latiflorus*. A study provided evidence showing that temperature changes help regulate the genes involved in cytokinin synthesis and signaling to some extent in *D. latiflorus* populations across elevation gradients. Also, temperature change influences the activity of enzymes responsible for sugar metabolism and, therefore, carbohydrate distribution and plant development [123]. Light control of phytohormone biosynthesis and transduction pathways has been described in *D. latiflorus*. This paper was able to determine that light intensity changes affect the synthesis of auxins and gibberellins, thus affecting the growth and development of plants [124].

Furthermore, light signals also control the patterns of genes that code for sugar transporters and enzymes that are concerned with sugar metabolism and, hence, influence carbon allocation to *D. latiflorus*. Water is one of the important physical factors that impacts phytohormonal activity and sugar content in *D. latiflorus*. Water shortage elicits changes in the synthesis of ABA that oversees the process of stomatal closure and water use efficiency under stress [89]. In addition, water stress affects sugar metabolism in relation to sucrose synthase and invertase enzymes that are responsible for the mobilization and storage of sugar. Hence, the nutrient status in soil affects the phytohormone signal transduction and sugar patterns in *D. latiflorus*. Some papers determined that nitrogen-dependent influences the concentration of auxins and cytokinins, which play the role of root development and nutrient acquisition effectiveness, respectively [27]. Furthermore, it was documented that phosphorus deficiency influences the metabolism of sugars due to the change in gene expression connected with sucrose and starch synthesis in the roots of *D. latiflorus*.

### 8.2. Biotic Interactions

Another biotic factor that may alter phytohormones and sugar status in *D. latiflorus* includes herbivory as well as symbiotic microbes. Herbivore-transported plant defenses involve the expression of the jasmonic acid (JA) signal transduction pathway recommended by shifting sugar accumulation to protective compounds [125]. Additionally, presumptive mutualistic mycorrhizal associations with fungi alter the auxin transport and the process of sugar transport in the roots of *D. latiflorus*, thus improving nutrient acquisition and stress tolerance. These studies are instrumental in elucidating the intricate environmental relations of *D. latiflorus* with regard to phytohormones/sugar metabolism and in emphasizing the need for the evaluation of the numerous ecological factors affecting the plant’s physiological processes and adaptations [126].

### 8.3. Ecological Significance and Ecosystem Services

In essence, there is little realized ecological value for this species, which is discerned from its ability to grow rapidly, versatility, and other functions within specific ecosystems. *D. latiflorus* has an essential function in ecosystems, being an indicator species in different biotopes and being involved in the processes of maintaining biological diversification. Apart from supporting and protecting soil from erosion, its root structure improves water penetrative capacities as well as nutrient circulation, thus improving the quality and fertility of the soil. Also, it is home to many species and is observed to be a source of food for various fauna, thereby supporting the diversification of species and ecosystems [127]. Thus, it is concluded that the high growth rate and perenniality of *D. latiflorus* enable us to consider this shrub as a promising species for increasing biomass productivity and effective carbon sequestration.

Utilizing the biomass of *D. latiflorus* would eliminate the release of greenhouse gases in the atmosphere, decrease the demand for fossil fuels, and support climate change adaptation. Further, *D. latiflorus*-based agroforestry systems also provide the potential for the improvement of ecosystem goods and services, livelihood diversification, and socio-economic rural development. Information on the molecular aspects related to the growth and development of *D. latiflorus* is critical for the proper management of the crop, increasing its stress tolerance and increasing biomass yield [19]. Auxin, cytokinin, gibberellin, and abscisic acid are significant phytohormones involved in diversified and proportional functions concerning the growth and development of *D. latiflorus* plants.

**Figure 4:**
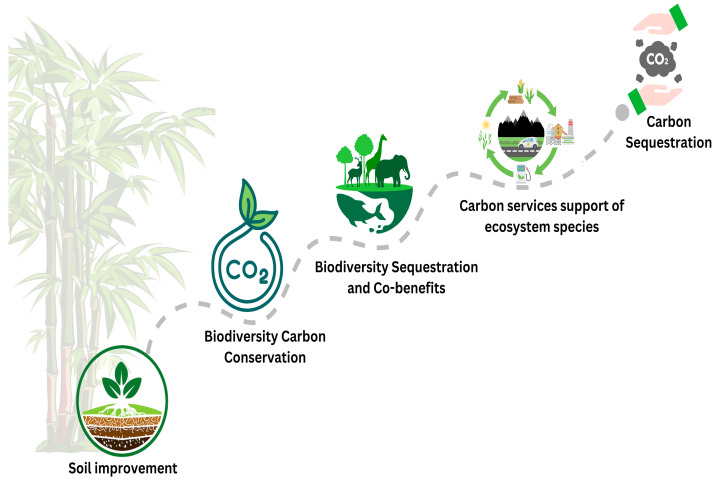
The ecological roles of *D. latiflorus* include its contribution to soil improvement, biodiversity conservation, and carbon sequestration.

Optionally, phytohormones regulate the dynamics of cell elongation and division and the stress reactions in *D. latiflorus* and change the expression of genes involved in these processes. Also, *D. latiflorus* relies on the regulation of sugar metabolism and carbohydrate partitioning in order to maintain its energy productivity and proper distribution of resources. *D. latiflorus* specifically regulates sugar moving, sucrose synthases, and inverses to guarantee the carbohydrate distribution between growth, storage, and defense pathways in contention with environmental changes. Understanding the molecular mechanisms that control sugar metabolism and distribution identifies key components of *D. latiflorus* that could help enhance its physiological processes and increase its ability to adapt to potential environmental changes (Figure 4). From a commercial perspective, the crosstalk between phytohormones and sugar metabolism supports rapid biomass production and stress tolerance, key traits for sustainable forestry and agroforestry practices. For example, gibberellins enhance stem elongation by increasing the flux of sugars through glycolysis, ensuring robust culm development, which is critical for industries such as construction and paper production. The ability to optimize these molecular pathways presents opportunities for improving bamboo yield and quality in commercial plantations.

## 9. Conclusions

*D. latiflorus*, the giant bamboo or timber bamboo, is arguably one of nature’s many works of art and one that will stand out due to its uniqueness. In the current review, we have uncovered the various aspects pertaining to *D. latiflorus* and presented the findings based on the applied biological and molecular framework, novel therapeutic uses, and the future of this plant. Recent studies of *D. latiflorus* have revealed that auxins promote culm elongation by activating cell wall-loosening enzymes. At the same time, abscisic acid mediates drought tolerance by regulating sucrose cleavage enzymes such as invertases. Although the role of phytohormones in general plant growth is well-established, research on their specific interactions with sugar metabolism in bamboo remains limited. The integration of phytohormones and sugar catabolism not only enhances the ecological resilience of *D. latiflorus* but also underpins its utility in commercial applications. This gap underscores the need for species-focused studies to validate and expand upon current hypotheses. Thus, summing up the exploration of *D. latiflorus*’s elaborate context and highlighting how this species might alleviate environmental issues and foster sustainable development goals, it is possible to conclude that this plant has enormous potential for various applications and further research. Further research on transcription factors like ARFs and MYBs and the role of PTMs in regulating hormonal signaling and metabolic processes is essential to unraveling the unique physiological mechanisms in *D. latiflorus*. This review uniquely integrates molecular and ecological perspectives on *D. latiflorus*. This review underscores the dynamic interplay between phytohormones and sugar metabolism in *D. latiflorus*, presenting a novel synthesis of molecular and ecological insights. Integrating species-specific regulatory networks with environmental and ecological factors offers a comprehensive framework for understanding the growth, stress resilience, and adaptability of this economically and ecologically valuable bamboo species. These findings lay a foundation for targeted breeding and management strategies aimed at improving the productivity and adaptability of *D. latiflorus* in response to global climate challenges.

## 10. Future Research Directions

Future research agendas should focus on further understanding the molecular aspects of the role of the environment in phytohormones and sugar metabolism in *D. latiflorus*. Combining omics methods, for example, transcriptomics, metabolomics, and proteomics, may give systematic information on genes, enzymes, and metabolites in different environments. The analysis of genotype by environment will increase knowledge about the genetic causes of geographical differences in *D. latiflorus* [19]. GWAS and QTL can pinpoint the genes and markers for adaptive traits that could help in improvement and conservation breeding. Further work should be conducted on the impact of such geographic differences of phytohormones and sugar catabolism in *D. latiflorus*. As a result, this study aims to contribute to the launching of the investigation of plant physiological adaptations regulating ecosystem processes, including carbon and nutrient cycling and ecosystem interactions with other species in the ecosystems that *D. latiflorus* dominates. In conservation efforts, *D. latiflorus* can be planted in biodiversity hotspots to enhance carbon sequestration while supporting local ecosystems. Its phytohormone-regulated root growth improves soil structure and prevents erosion, providing a stable habitat for native plant and animal species.

Evaluating the phytohormones and SUGs and their effects in relation to climate change on *D. latiflorus* is crucial for predicting the species’ reaction and applying them to management and conservation. Climate modeling techniques can forecast climate change for future years and assess *D. latiflorus* populations’ ability to adapt to climate change. Knowledge of the spatial patterns in phytohormones and sugar catabolism in *D. latiflorus* is crucial for understanding its responses to environmental gradients and predicting the species’ vulnerability under conditions of global change. To extend the current knowledge of plant adaptation and ecosystems’ responses, researchers must investigate how environmental factors influence plant traits and ecosystem dynamics; it is recommended to follow future research directions that expound on the mechanistic information, genotype–environment interactions, ecosystem-level responses, and climate change effects.

Future research on *D. latiflorus* should focus on several critical areas to advance our understanding of the phytohormone–sugar interplay and its applications. One key direction is exploring the molecular crosstalk between phytohormones and sugar metabolism, particularly the roles of transcriptional regulators and signaling pathways in mediating plant adaptation and stress resilience. While the influence of phytohormones on sugar metabolism is well-documented, the reciprocal effects of sugar metabolism on phytohormones, especially under stress conditions, remain underexplored. Another important area is the geographic variation in phytohormone levels and sugar metabolism. High-throughput techniques, such as genome-wide association studies (GWAS) and metabolomics, could be employed to identify genetic and metabolic adaptations to diverse environmental conditions. These approaches may reveal critical genes and pathways responsible for the environmental plasticity of *D. latiflorus*, offering insights into its ecological success.

Climate change presents additional challenges and opportunities for future research. Investigating how shifting environmental conditions, such as temperature fluctuations and water scarcity, affect the phytohormone–sugar interplay in *D. latiflorus* could guide its use in sustainable agroforestry systems. For instance, understanding its drought tolerance mechanisms may inform breeding strategies aimed at enhancing resilience in other economically important bamboo species. Translating this fundamental knowledge into practical applications remains a priority. Future studies should evaluate breeding techniques and management practices to improve stress tolerance, biomass productivity, and carbon sequestration capacity in *D. latiflorus*. This research could significantly contribute to sustainable forestry, biodiversity conservation, and climate change mitigation.

## Figures and Tables

**Table 1:** Overview of Phytohormones in *Dendrocalamus latiflorus.*

Phytohormone	Role in *D. latiflorus*	Biosynthesis	Key Functions	Recent Studies
Auxins	Cell elongation, root development, culm elongation	Shoot apices and young leaves	Promotes cell elongation, root initiation, stem growth, fruit development	[39]
Cytokinins	Cell division, shoot development, delays senescence	Roots	Promotes cell division shoot formation, delays leaf senescence, nutrient mobilization	[40]
Gibberellins	Stem elongation, seed germination, internode elongation	Plastids	Promotes stem elongation, seed germination, flowering	[24]
Abscisic Acid (ABA)	Stress response, stomatal closure, seed dormancy	Carotenoid precursors in plastids	Regulates stress responses, stomatal closure, seed dormancy	[41]
Ethylene	Fruit ripening, leaf senescence, stress responses	Methionine via SAM and ACC	Regulates fruit ripening, leaf abscission, and responses to biotic/abiotic stresses	[33]
Brassinosteroids	Cell expansion, stress tolerance, vascular differentiation	Campestral	Enhances cell expansion, stress tolerance, and vascular differentiation	[42]

**Table 2:** Summary of phytohormone biosynthesis, transport, and signaling pathways in *Dendrocalamus latiflorus*. This table outlines the key processes involved in phytohormone production, movement, and signaling within the plant, based on recent studies.

Phytohormone	Biosynthesis Pathway	Transport Mechanism	Signaling Pathway	References
Auxins	Synthesized from tryptophan via the indole-3-pyruvic acid and tryptamine pathways	Polar auxin transport (PAT) using PIN and AUX/LAX proteins	Perceived by TIR1/AFB receptors, leading to AUX/IAA degradation and ARF activation	[55]
Cytokinins	Synthesized in roots from adenine derivatives via the isopentenyl transferase (IPT) pathway	Transported via xylem	Binds to AHK2/AHK3 receptors, phosphorylates HPs, and activates RRs	[56]
Gibberellins	Produced in plastids via the methylerythritol phosphate (MEP) pathway involving GA20-oxidase and GA3-oxidase	Transferred through phloem and xylem	Binds to GID1 receptors, leading to DELLA protein degradation and growth activation	[57]
Abscisic Acid	Synthesized in plastids from carotenoid precursors via the 9-cis-epoxycarotenoid cleavage pathway	Transported through xylem and phloem	Interacts with PYR1/PYL/RCAR, inhibits PP2C, activates SnRK2, and phosphorylates ABA-responsive genes	[58]
Ethylene	Derived from methionine through S-adenosylmethionine (SAM) and 1-aminocyclopropane-1-carboxylic acid (ACC)	Diffuses through plant tissues	Perceived by ETR1 receptors, activates EIN3 transcription factors	[59]
Brassinosteroids	Derived from campesterol via BRASSINOSTEROID-6-OXIDASE (BR6OX) enzymatic transformations	Transported locally or short distances	Recognized by BRI1 and BAK1 receptors, activates BZR1 transcription factors	[60]

**Table 3:** Key enzymes in sugar catabolism in *D. latiflorus.*

Enzyme	Pathway	Function	Key Roles in *D. latiflorus*	Recent Studies
Invertase	Sucrose Metabolism	Hydrolyzes sucrose into glucose and fructose	Provides glucose and fructose for metabolic processes	[80]
Sucrose Synthase (SuSy)	Sucrose Metabolism	Converts sucrose and UDP into UDP-glucose and fructose	Supports cellulose and callose synthesis during cell wall formation	[81]
Hexokinase (HXK)	Glycolysis	Phosphorylates glucose to glucose-6-phosphate	Initiates glycolysis, glucose sensing	[82]
Alpha-Amylase	Starch Degradation	Hydrolyzes alpha-1,4-glycosidic bonds in starch	Produces maltose and glucose from starch	[83]
Beta-Amylase	Starch Degradation	Cleaves non-reducing end of starch	Produces maltose from starch	[84]
Glucanotransferase	Starch Degradation	Transfers glucosyl units	Modifies starch structure	[85]
Debranching Enzyme	Starch Degradation	Cleaves alpha-1,6-glycosidic bonds	Facilitates complete starch degradation	[86]

**Table 4:** Interplay between phytohormones and sugar catabolism in *D. latiflorus.*

Phytohormone	Influence on Sugar Metabolism	Sugar’s Influence on Phytohormones	Recent Studies
Auxins	Modulates sugar transport and metabolism	Sugars affect auxin biosynthesis and transport	Mishra, Sharma and Laxmi [43]
Cytokinins	Influences sugar transporters and enzymes	Sucrose promotes cytokinin biosynthesis	Wang, et al. [104]
Gibberellins	Regulates glycolysis and TCA cycle genes	High sugar levels suppress GA biosynthesis	Lan, et al. [95]
Abscisic Acid	Enhances sucrose cleavage under stress	Glucose modulates ABA signaling pathways	Liao, et al. [105]
Ethylene	Regulates glycolysis and TCA cycle genes	Sugars influence ethylene production and signaling	Li, et al. [106]
Brassinosteroids	Enhances glycolysis and TCA cycle genes	Sugars modulate brassinosteroid signaling pathways	Zheng, et al. [107]

**Table 5:** Geographic variation in phytohormones and sugar metabolism in *D. latiflorus*.

Geographic Location	Phytohormone Variation	Sugar Metabolism Variation	Environmental Influence	Recent Studies
High Altitude	Enhanced cytokinin and gibberellin	Increased sucrose synthesis	Low-temperature adaptation	[118]
Low Altitude	Higher auxin and ethylene levels	Increased starch degradation	Warmer climate adaptation	[119]
Water-Limited	Elevated abscisic acid levels	Enhanced sucrose cleavage, altered starch metabolism	Drought stress adaptation	[120]
Nutrient-Rich Soil	Balanced hormone levels	Efficient sugar transport and metabolism	Nutrient availability	[121]

## Data Availability

No new data were created or analyzed in this study. Data sharing is not applicable to this article.
